# Enhancement of the ‘tractor-beam’ pulling force on an optically bound structure

**DOI:** 10.1038/lsa.2017.135

**Published:** 2018-01-12

**Authors:** Jana Damková, Lukáš Chvátal, Jan Ježek, Jindřich Oulehla, Oto Brzobohatý, Pavel Zemánek

**Affiliations:** 1 Institute of Scientific Instruments of the CAS, v.v.i., Královopolská 147, Brno 612 64, Czech Republic

## Abstract

Recently, increasing attention has been devoted to mastering a new technique of optical delivery of micro-objects tractor-beam’^1, 2, 3, 4, 5, 6, 7, 8, 9^. Such beams have uniform intensity profiles along their propagation direction and can exert a negative force that, in contrast to the familiar pushing force associated with radiation pressure, pulls the scatterer toward the light source. It was experimentally observed that under certain circumstances, the pulling force can be significantly enhanced^6^ if a non-spherical scatterer, for example, a linear chain of optically bound objects^10, 11, 12^, is optically transported. Here we demonstrate that motion of two optically bound objects in a tractor beam strongly depends on theirs mutual distance and spatial orientation. Such configuration-dependent optical forces add extra flexibility to our ability to control matter with light. Understanding these interactions opens the door to new applications involving the formation, sorting or delivery of colloidal self-organized structures.

The rapidly growing domain of optical micro-manipulation techniques and applications^13^ has been recently enriched by the experimental demonstration of an optical tractor beam^6^, which is based on a counter-intuitive pulling of object toward the source of radiation. The pulling force drags objects against the overall photon flow (given by the resultant wave vector **k**) only if the majority of incident photons are scattered close to the direction of the wave vector **k**. However, a much more common pushing force is proportional to radiation pressure and dominates if the photons are scattered off the direction of the wave vector **k** (for example, back-reflection). The pulling force arises if one uses appropriate combinations of interfering beams^1, 6^ or plane waves^3, 7, 14^ of a tailored amplitude and phase, single Bessel^3, 4^ or solenoid beams^2^. Alternatively, one can take advantage of structured media supporting backward waves^15^, of gain media^16^ or suitably designed chiral metamaterials^17^ or an object floating on a liquid-air interface^8^. There are also conceptually different ‘active’ tractor beams that are based on ‘optical-conveyor’ belts^18, 19, 20^, where the moving gradient of the optical intensity (for example, moving interference fringe) is essential for the object delivery.

To verify the concept of the tractor beam experimentally, we recently designed^6, 21^ a configuration that uses a wide Gaussian beam retro-reflected on a dielectric mirror, as illustrated in Figure 1a. The pulling force drags objects along the *z* axis, against the overall photon flow direction given by **k**=**k**_1_+**k**_2_. On the basis of the principle of action and reaction, the transfer of momentum leads to this backward movement of the objects^3, 6, 14^ if the photons are dominantly scattered toward the *z* axis and in the geometry of Figure 1 propagate against the *z* axis. However, the region where the pulling or pushing force predominates is limited by a restorative gradient force **F**_grad_, which is proportional to the gradient of the lateral Gaussian beam profile. This gradient force attracts objects to the center of the beam at *z*=0, where the beam intensity is the highest. Therefore, once the object has reached a stable position, due to the balance between gradient and pulling/pushing forces, its deviation *z*>0 (*z*<0) from the beam central position reveals the magnitude of the pulling (pushing) force.

Tractor-beam properties for a single particle have been already comprehensively analyzed^6^. However, it was experimentally observed that under certain circumstances, the pulling force can be significantly enhanced^6^ if a linear chain of optically bound objects^10, 11, 12^ is optically transported. Since this pulling force enhancement has not been sufficiently explained yet, we focus here on the case of two optically self-arranged and bound objects, keeping the same experimental parameters, i.e., polystyrene spheres with a diameter of 820 nm and beam incident angle *α*<4 ° see Figure 1a. We set the polarization of the incident beam to be perpendicular to the plane of incidence (S-polarization), i.e. this polarization causes an isolated particle to be pulled against the photon flow. We studied the behavior of the particle pair relatively far (~50 μm) from the surface of the dielectric mirror, where the influence of the reflected scattered field on the particle self-arrangement is very weak.

To track the motion of each particle in all three dimensions, we employed holographic deconvolution video microscopy^22^. Therefore, we were able to reveal the dependence of the overall behavior of a pair of particles on the relative spatial configuration of the pair. Processing all of the hologram video frames (Figure 1b and Supplementary Media 1), we identified that the predominant motion of particles is in the *z* direction and negligible in the *x* direction. Surprisingly, despite the beam parameters remaining unchanged, the particle pair was pushed by the laser beam (indicated by region *i–ii* in Figure 1c) and subsequently pulled against the beam propagation. Figure 1c reveals remarkable changes in the spatial configuration of the particles occurring during the motion, mainly in the *y* direction. Qualitatively, the measurements demonstrate that the direction of the pair motion depends strongly on the internal configuration and orientation of the pair.

A brief intuitive explanation of the optical binding process and direction of the particle pair motion is based on understanding the modification of the tractor beam field by particles themselves. The first particle scatters the incident tractor beam and creates lobes with a higher optical intensity, where the second particle is attracted into due to the gradient force arising in such intensity profile. The optical binding between particles restricts the inter-particle distance to several stable configurations of the particle pair shown in Figure 2a and b. However, due to the non-zero angle of incidence *α*, the particles generally do not reach the maximal intensity of the lobe and stay localized aside, which consequently induces their motion because they are mutually propelled by the gradient force toward the high-intensity part of the running ahead lobe (Figure 2c–h, Supplementary Media 2). In addition, particles are attracted to the tractor beam standing wave fringes. However, due to the Brownian motion, particles can overcome the potential barrier of this standing wave and jump to the neighboring fringes and thus change their height above the mirror as well as the inter-particle distance (*y* coordinate).

At the stable configuration of optically bound particles, their inter-particle distance does not change; thus, the same total force acts on both particles in the considered over-damped system along the *z* axis: *F*_1*,z*_=*F*_2*,z*_. The total optical force *F*_2*,z*_ acting on one particle in the pair can be written as *F*_2*,z*_=*F*_isol2*,z*_+*F*_int2*,z*_, where *F*_isol2*,z*_ acts on the isolated particle if the first particle is absent (denoted by unfilled black arrows in Figure 2b), and the interaction force *F*_int2*,z*_ exists due to the presence of the first particle (filled black arrows in Figure 2b). Since we consider particles 1 and 2 of equal properties placed in an almost homogeneous incident beam (with respect to the inter-particle distance), *F*_isol1*,z*_=*F*_isol2*,z*_; thus, *F*_int1*,z*_=*F*_int2*,z*_. For particles that are optically bound along the *y* axis, that is, following the blue lobe in Figure 2b, the interaction force *F*_int2*,z*_ directs to the lobe maximal intensity, which is always opposite to the direction of the force acting on the isolated particle *F*_isol2*,z*_. Since the magnitude of the interaction force depends strongly on the inter-particle distance, while the force on the isolated particles remains constant, we observe that the total force switches between pushing and pulling when the inter-particle distance increases (Figure 3a and Supplementary Media 2).

A similar behavior can be observed for the particles that are optically bound in the second and third lobe (red dots in Figure 2b), which corresponds to our experimental observation presented in Figure 1. However, here, the stable particle position jumps from the second to the third lobe with increasing inter-particle distance and, in contrast to the previous case, the interaction force changes its sign and remains the leading force (|*F*_int2*,z*_|>|*F*_isol2*,z*_|) in the investigated region. This change from pushing to pulling occurs because the particles in the second and third lobes are on opposite sides with respect to the lobe intensity maximum and thus are attracted in opposite directions (Figure 2c and d, and Supplementary Media 2). The plots in Figure 2e–h prove that the rigorous total force acting on the particle (full black) is very close to the profile (thin green/red), which originates purely from the gradient of the optical intensity along the *z* axis, where the interaction force predominates. Thus, even the rigorous calculations support the intuitive conclusion that the particles ‘surf’ along the *z* axis on the slope of the traveling intensity scattering pattern (lobe) of the other particle. Considering the other lobes, the force *F*_isol2*,z*_ is the leading one, and the total force does not change its direction and follows the direction of the force acting on an isolated particle. The same intuitive picture works for the P-polarized incident beam, as Supplementary Media 3 demonstrates.

Since the particles move in a viscous medium, their motion is over-damped, and their velocity is directly proportional to the total force acting on them. Each stable configuration of the particle pair is propelled with a different speed and in a different direction, as the lengths and colors of the arrowhead marks demonstrate in Figure 3a, respectively. If the experimental and theoretical data in Figure 3a are compared, they perfectly agree in the direction of the particle pair motion (the color of the experimental curve indicates the direction of motion). Indeed, there are configurations (mainly in the first three scattering lobes) where the direction of motion of the particle pair is either opposite to that of an isolated particle (see orange arrowhead marks) or parallel, but its magnitude is strongly enhanced. These cases correspond to the situation when the interaction force, arising from the gradient of the lobe intensity profile, predominates and drives the behavior of the system, as we explained in Figure 2b. Quantitative comparison of velocities of the pair versus the single isolated particle in Figure 3b gives persuading agreement between the experimental data and theoretical predictions. Moreover, Figure 3b also proves that the hydrodynamic interaction between particles does not increase the pair velocity significantly, and the observed phenomena are solely of optical origin.

As Figure 3c demonstrates, under optimal angle of incidence and beam polarization, the pulling and pushing forces on the particle pair (directly proportional to the plotted velocities) can be enhanced by an order of magnitude compared with a single isolated particle. The pulling force acting on the particle pair appears even for the P-polarized tractor beam, where the single particle is only pushed. Thus, our numerical simulations, which are carried out in a wide range of tractor beam incident angles and two polarizations, reveal that the direction of particle pair motion is given by the pair configurations rather than by behavior of an isolated particle at the same conditions.

Approaching the bottom mirror with the pair of optically bound particles^6, 21^ makes the interaction between particles via the scattered light even more complex due to the reflection of the re-scattered field; see Figure 4. In contrast to the previous case, the scattered field consists of three contributions: the field scattered on particle 1 placed at the coordinate origin, its mirror image 1’, and the mirror image of the second particle 2’, as Figure 4c explains. Thus, one can expect that the behavior of the particle pair near the mirror differs from the geometry studied above. Following the same symbolism as in Figure 3a, the optical intensities around particle 2 are shown in Figure 4b at three different distances of the particle from the dielectric mirror. The steady-state positions of the particle pair are created preferentially very close to the extremities of the intensity profile, and similar to the previous case, the numerically determined particle pair velocities show the complex nature of this phenomenon. In Figure 4d, experimental observations reveal that the particles are aligned parallel with the mirror (*y*_2_=*y*_1_), and the pulling force exists for the S-polarized beams as well as for the P-polarized beams for more distant particles. Such behavior coincides with the last row of theoretical calculations in Figure 4b, where the yellow and green shaded areas indicate the experimentally measured separations between the particles.

In summary, we have shown that optical transport of micro-particles in tractor beams can be up to an order of magnitude enhanced via optically mediated self-arrangement of the micro-particles. Such optically bound structures can exhibit a rich dynamical behavior, which critically depends on their spatial arrangement and easily experimentally controllable parameters of the beam (for example, polarization and angle of incidence). In addition to rigorous calculations, we introduced an intuitive picture of the mechanism of pulling and pushing of the pair of particles that resembles particles ‘surfing’ on the slope of the scattered intensity pattern formed by and traveling with the other particle. Understanding the underlying physical mechanisms opens new opportunities in a controllable light-driven self-organization, sorting and transport of colloidal matter.

## Methods

### Experimental tractor-beam setup

**Figure 1:**
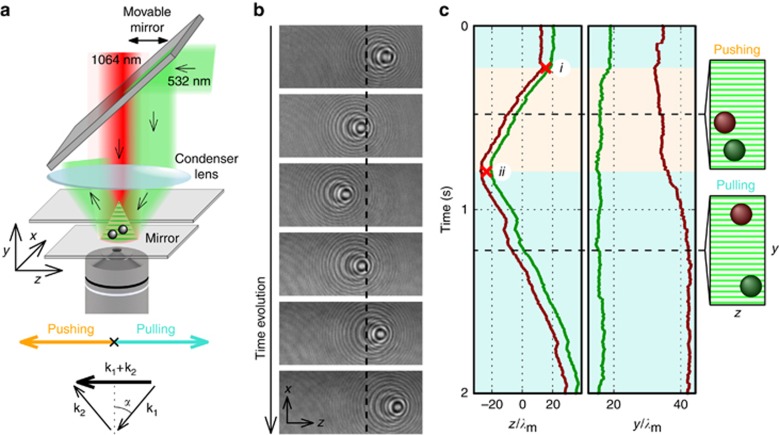
(**a**) Schematic illustration of the experimental optical setup. The 820-nm polystyrene spheres are optically bound in a tractor beam (*λ*_0_=532 nm, incident angle *α*=2.15 °, S-polarization, green beam) and illuminated using a 1064-nm laser (red beam), which is utilized for holographic video microscopy. The wavevector diagram illustrates direction of the incident (**k**_**1**_) and reflected (**k**_**2**_) beam. (**b**) Typical sequence of recorded hologram images of two optically bound particles with the subtracted hologram background (see Supplementary Media 1 for the full movie). Dashed line marks the beam center *z*=0. (**c**) Time dependence of positions of two optically bound particles in *z* and *y* axis shown in **b**, where *λ*_m_=400 nm is the tractor beam wavelength in an aqueous medium, and the beam is centered at *z*=0. The orange or turquoise background corresponds to the pushing or pulling regime, respectively. The right images visualize the relative configurations of the particles at two selected moments marked by dashed lines. Red crosses highlight important points that interconnect experimental data with numerical calculations shown in Figure 3a. For more details, see Methods and Supplementary Information.

Experimental optical setup is schematically illustrated in Figure 1a. The tractor beam is formed via retro-reflection of a wide Gaussian beam (VERDI V18, Coherent, Inc., *λ*_0_=532 nm, *w*_0_=35 μm, *P*=3.5 W in the sample plane) at a dielectric mirror, which is fabricated via electron beam evaporation (alternating SiO_2_ and TiO_2_ layers) and supports the colloidal suspension of 820-nm polystyrene spheres (Duke Scientific Corporation, Palo Alto, CA, USA). The beam polarization is controlled using a half-wave plate, and the angle of incidence is defined by the position of a movable mirror above the condenser lens of a 150-mm focal length.

### Particle tracking

Positions of the optically bound micro-particles were precisely determined in all three spatial dimensions via the holographic video microscopy technique^22, 23^. For that purpose, another infrared laser beam (ADLAS GmbH and Co., *λ*_0_=1064 nm) is introduced in the setup only to illuminate the particles, with no significant mechanical effects due to its much lower intensity. The field scattered by the particle interferes downstream with the incident beam field *E*_0_. The distribution of |*E*|^2^— a hologram— in the planes transverse to the beam path is typically patterned with circular fringes around particle positions. The hologram is magnified by the objective lens (Olympus UPlanFl 100 × NA1.3 oil immersion) and projected on a fast CCD camera (Basler acA640–750 μm). The recorded holograms were normalized using a background image, obtained via median-filtering each pixel intensity over sufficient number of frames. To compensate for intensity drifts, the entire video is boxed into shorter sequences, which were subjected to filtering.

The scattered field in the volume above the objective focal plane is reconstructed from the hologram using the Rayleigh-Sommerfeld back-propagation method^23^, followed by deconvolution^22^ to remove artifacts and twin images. Then, the 3D positions of the particles are determined as the centroids of the brightest areas in the reconstructed scattered field.

### Numerical methods (simulations)

The optical forces acting on individual spheres were calculated via the Maxwell stress tensor integrals^24^ employing multipolar expansion of fields provided by the multi-particle Mie theory^25^. During the calculation of optical forces, we solve the electromagnetic scattering problem in a self-consistent way, that is, we take into account enough reflections to achieve numerical convergence. The 820-nm diameter of the sphere, its refractive index *n*_p_=1.59, and water refractive index *n*_m_=1.33, imply the expansion order cutoff *N*_max_ ~20 to ensure convergence. The ideal plane-wave tractor beam is considered in our numerical model, which is valid close to the Gaussian beam axis.

Each sphere in the pair remains tightly bound to the centers of their respective fringe, except for the very closest configurations, where the near fields shift the centers by a little fraction of *λ*_0_ in *y*. This was verified numerically for deterministic trajectories of all particle pairs that were initially displaced from the expected steady configuration. Methods of variable step and order for the stiff problems (equivalent to Matlab procedures ode15s and ode113) were used for the numerical integration of the over-damped equations of motion. An overall linear stability^26^ in the co-moving frame was reached.

## Author contributions

OB and PZ conceived and supervised the project, LC provided theoretical analysis, JD, JO, OB and JJ performed and analyzed the experiments. All authors contributed to the preparation of the manuscript.

## Figures and Tables

**Figure 2:**
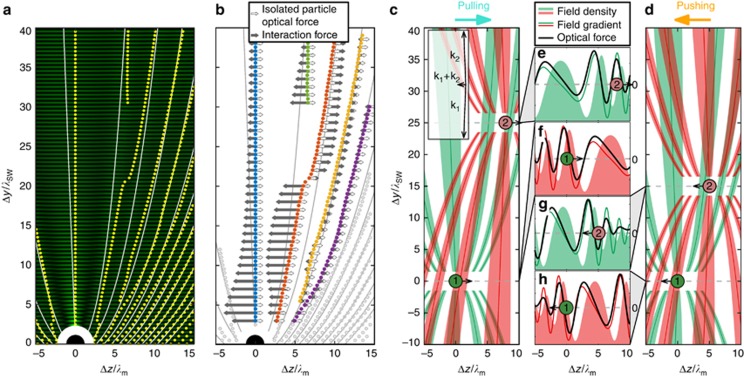
(**a**) Stable configurations of the particle pair. The first particle is placed at the origin of the coordinate system (big black dot), and the steady-state positions of the second particle are marked by yellow dots. The calculated optical intensity is shown as the background. Due to the interference between the incident and reflected beam, the horizontal interference fringes are formed. As the result of the interference between the incident and scattered light, the intensity lobes are formed, and their maxima are highlighted by thin solid white curves. The *y* axis is normalized to the length of two fringes *λ*_SW_=*λ*_m_/cos*α*. (**b**) The total force *F*_2*,z*_ acting upon the second particle can be written as *F*_2*,z*_=*F*_isol2*,z*_+*F*_int2*,z*_, where the force *F*_isol2*,z*_ acts on the particle if the first particle is absent (denoted by unfilled black arrows), and *F*_int2*,z*_ denotes the interaction force due to the presence of the first particle (filled black arrows). The open gray circles denote stable configurations that were not studied here. (**c** and **d**) The direction of the interaction force *F*_int*,z*_, which determines the total tractor force, is given by the position of particles with respect to the scattering lobe maxima. (**c**) In the formed optically bound structures, the particles are localized on the left-hand side of the lobe maxima and pulled toward the runaway lobe maximum. Analogously for pushing in **d**, where the bound particles are pushed toward the lobe intensity maximum. The color of the lobe pattern follows the color of the particle. (**e**–**h**) Plots compare the calculated optical intensity profiles along the *z* axis (filled green/red), their gradients along the *z* axis (thin green/red) and the optical forces (full black) acting on the particular particle. The particles are drawn at the positions corresponding to the stable equilibrium configuration of the particle pair. See Supplementary Media 2 for more details. Parameters of the calculations: *λ*_m_=400 nm, incident angle of the S-polarized tractor beam *α*=2.15 °, polystyrene particles with an 820-nm diameter.

**Figure 3:**
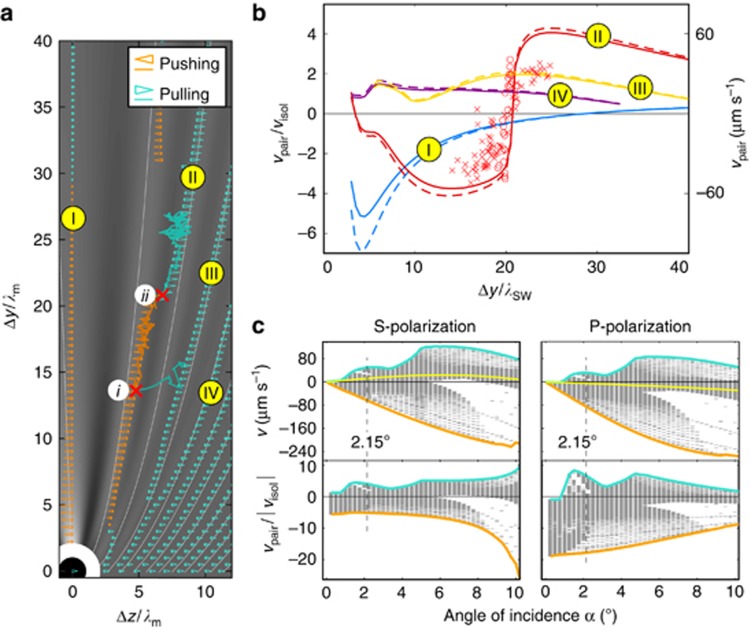
(**a**) Comparison of the measured trajectories with the calculated lobes and particle velocities. The background is proportional to the calculated optical intensity (shown in log scale) of the total electric field averaged in *y* over one fringe length *λ*_SW_/2. The white thin curves have the same meaning as in Figure 2a. Calculated velocities of the particle pair along the *z* axis are encoded in the length of the triangles (proportional to 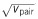). For comparison, the velocity of an isolated particle *v*_isol_ is shown at the center of particle 1 placed at the origin of the system of coordinates. The solid zigzag curves represent experimental results, and their colors encode the direction of the particle pair motion (see Supplementary Information for more experimental results). (**b**) Comparison of the particle pair velocity, *v*_pair_, and the relative pair velocity, *v*_pair_/*v*_isol_, for the selected configuration groups that are distinguished here by colors (the same as in Figure 2b) and denoted by Roman numerals as in Figure 3a. Different markers correspond to different experimental data sets performed under the same experimental conditions (incident angle of the S-polarized tractor beam *α*=2.15°). The curves show the calculated velocities of the particle pair if the hydrodynamic interaction between the particles is (dashed) and is not (full) added to the theoretical model (see Supplementary Information for more details). (**c**) Comparisons of the calculated velocities, *v*_pair_, of the particle pair along the *z* axis for different angles of incidence *α* and polarizations of the incident beam. Each horizontal gray segment corresponds to one steady-state configuration of the pair for considered *α* and polarization calculated in the same region of the *y* and *z* axis as in Figure 3a. The yellow curve in the top plots denotes the velocity, *v*_isol_, of a single isolated particle in the tractor beam, and the bottom plots reveal the relative enhancement of the pulling (but also pushing) force (proportional to *v*_pair_/*v*_isol_) in particular configurations of the incident beam and the particle pair. The velocity extremes are highlighted with the thick curves, pulling and pushing is encoded in the curve color as above. We considered the incident power density 1.8 mW μm^−2^ in all theoretical results presented in this figure.

**Figure 4:**
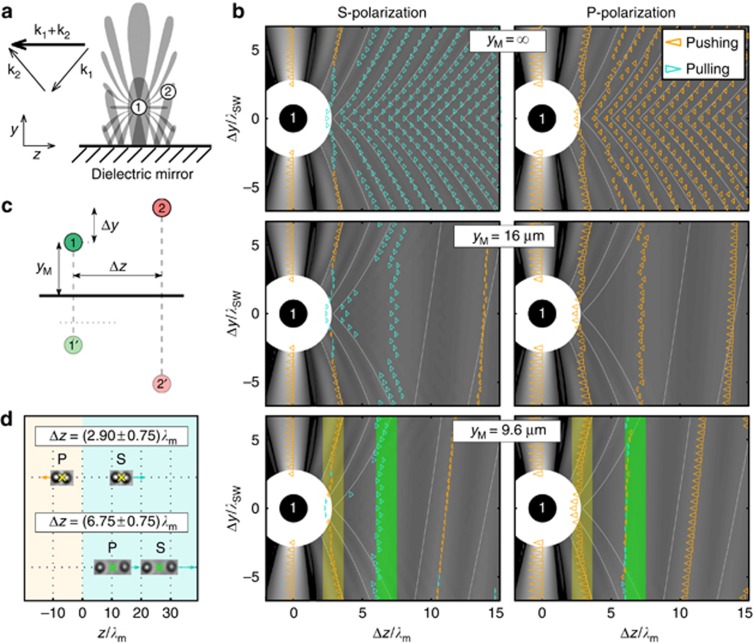
(**a**) Schematic configuration of a pair of optically bound particles near the dielectric mirror and illustration of the intensity lobes created by interference of the tractor beam and the field scattered by particle 1. (**b**) Calculated velocities (triangle length proportional to the square root of the pair speed) of stable 820-nm sphere pairs for the S- and P-polarized beams incident at an angle of *α*=2.82°. The distance *y*_M_ of particle 1, which is placed at the coordinate origin, from the dielectric mirror is ∞, 16 and 9.6 μm (close to the experimental value). The background denotes the electric field density calculated as in Figure 3a, the scattered field is taken as the sum of the field scattered by particle 1, its mirror image 1’, and the mirror image of the particle 2’, shown in **c**. The green and yellow shadings correspond to the smaller and larger observed inter-particle distance in **d**, respectively. (**d**) Experimental observations of the stable positions of the centers of particle pairs near the dielectric mirror for the S- and P-polarized tractor beams.

## References

[bib1] Sukhov S, Dogariu A. On the concept of ‘tractor beams’. Opt Lett 2010; 35: 3847–3849. 21082017 10.1364/OL.35.003847

[bib2] Lee SH, Roichman Y, Grier DG. Optical solenoid beams. Opt Express 2010; 18: 6988–6993. 20389718 10.1364/OE.18.006988

[bib3] Chen J, Ng J, Lin ZF, Chan CT. Optical pulling force. Nat Photonics 2011; 5: 531–534.

[bib4] Novitsky A, Qiu CW, Wang HF. Single gradientless light beam drags particles as tractor beams. Phys Rev Lett 2011; 107: 203601. 22181730 10.1103/PhysRevLett.107.203601

[bib5] Ruffner DB, Grier DG. Optical forces and torques in nonuniform beams of light. Phys Rev Lett 2012; 108: 173602. 22680864 10.1103/PhysRevLett.108.173602

[bib6] Brzobohatý O, Karásek V, Šiler M, Chvátal L, Čižmár T et al. Experimental demonstration of optical transport, sorting and self-arrangement using a ‘tractor beam’. Nat Photonics 2013; 7: 123–127.

[bib7] Sukhov S, Dogariu A. Negative nonconservative forces: optical ‘tractor beams’ for arbitrary objects. Phys Rev Lett 2011; 107: 203602. 22181731 10.1103/PhysRevLett.107.203602

[bib8] Kajorndejnukul V, Ding WQ, Sukhov S, Qiu CW, Dogariu A. Linear momentum increase and negative optical forces at dielectric interface. Nat Photonics 2013; 7: 787–790.

[bib9] Shvedov V, Davoyan AR, Hnatovsky C, Engheta N, Krolikowski W. A long-range polarization-controlled optical tractor beam. Nat Photonics 2014; 8: 846–850.

[bib10] Burns MM, Fournier JM, Golovchenko JA. Optical matter: crystallization and binding in intense optical fields. Science 1990; 249: 749–754. 17756787 10.1126/science.249.4970.749

[bib11] Dholakia K, Zemánek P. *Colloquium*: gripped by light: optical binding. Rev Mod Phys 2010; 82: 1767–1791.

[bib12] Demergis V, Florin EL. Ultrastrong optical binding of metallic nanoparticles. Nano Lett 2012; 12: 5756–5760. 23035835 10.1021/nl303035p

[bib13] Gao DL, Ding WQ, Nieto-Vesperinas M, Ding XM, Rahman M et al. Optical manipulation from the microscale to the nanoscale: fundamentals, advances, and prospects. Light Sci Appl 2017; 6: e17039. 10.1038/lsa.2017.39 PMC6062326 30167291

[bib14] Sáenz JJ. Optical forces: laser tractor beams. Nat Photonics 2011; 5: 514–515.

[bib15] Salandrino A, Christodoulides DN. Reverse optical forces in negative index dielectric waveguide arrays. Opt Lett 2011; 36: 3103–3105. 21847174 10.1364/OL.36.003103

[bib16] Mizrahi A, Fainman Y. Negative radiation pressure on gain medium structures. Opt Lett 2010; 35: 3405–3407. 20967081 10.1364/OL.35.003405

[bib17] Fernandes DE, Silveirinha MG. Optical tractor beam with chiral light. Phys Rev A 2015; 91: 061801.

[bib18] Čižmár T, Garcés-Chávez V, Dholakia K, Zemánek P. Optical conveyor belt for delivery of submicron objects. Appl Phys Lett 2005; 86: 174101.

[bib19] Čižmár T, Kollárová V, Bouchal Z, Zemánek P. Sub-micron particle organization by self-imaging of non-diffracting beams. New J Phys 2006; 8: 43.

[bib20] Ruffner DB, Grier DG. Optical conveyors: a class of active tractor beams. Phys Rev Lett 2012; 109: 163903. 23215079 10.1103/PhysRevLett.109.163903

[bib21] Brzobohatý O, Čižmár T, Karásek V, Šiler M, Dholakia K et al. Experimental and theoretical determination of optical binding forces. Opt Express 2010; 18: 25389–25402. 21164887 10.1364/OE.18.025389

[bib22] Dixon L, Cheong FC, Grier DG. Holographic deconvolution microscopy for high-resolution particle tracking. Opt Express 2011; 19: 16410–16417. 21935004 10.1364/OE.19.016410

[bib23] Lee SH, Grier DG. Holographic microscopy of holographically trapped three-dimensional structures. Opt Express 2007; 15: 1505–1512. 19532383 10.1364/oe.15.001505

[bib24] Farsund Ø, Felderhof BU. Force, torque, and absorbed energy for a body of arbitrary shape and constitution in an electromagnetic radiation field. Phys A Stat Mech Appl 1996; 227: 108–130.

[bib25] Mackowski D The extension of Mie theory to multiple spheres In: Hergert W, Wriedt T eds. The Mie Theory. Berlin, Heidelberg: Springer; 2012.

[bib26] Simpson SH, Hanna S. First-order nonconservative motion of optically trapped nonspherical particles. Phys Rev E 2010; 82: 031141. 10.1103/PhysRevE.82.031141 21230059

