# Supplementary material for: Enhancement of the ‘tractor-beam’ pulling force on an optically bound structure
Source: Light Sci Appl. 2018 Jan 12;7:17135–. doi: 10.1038/lsa.2017.135 (PMC6107043; doi:10.1038/lsa.2017.135)
Supplement: Supplementary Information [file lsa2017135x1.pdf]

Supplementary Information: Enhancement of the "tractor-beam" pulling force on an optically bound structure

Jana Damková, Lukáš Chvátal, Jan Ježek, Jindřich Oulehla, Oto Brzobohatý and Pavel Zemánek*

Institute of Scientific Instruments of the CAS, v.v.i., Královopolská 147, 612 64 Brno, Czech Republic

1 Comparison of the measured trajectories with theoretical calculations

In Figures S1 and S2 we compare more experimental and theoretical results related to the behavior of a particle pair optically bound in the tractor-beam. The measured trajectories marked with zigzag curves are compared with the calculated velocities of the stable optically bound particle pairs. We observed very good agreements between inter-particle distances and calculated stable positions. These results follow the conclusions of the main text regarding the particle motion in the second/third scattering lobe (Figures S1a-c). Here the interaction force due to the presence of the first particle rules the particle movement and thus, there are significant changes in the direction of particle pair motion. Figure S1d demonstrates such reversal of particle pair motion in different lobe, too. In a large number of measurements, the particle pair was optically bound in higher-order scattering lobes, where the interaction force was not dominant and thus the pair was pulled by the S-polarized tractor-beam (see Figure S2) as an isolated particle. Even here the inter-particle distances correspond to the calculated stable positions and the second particle was moving generally close to the scattering lobe maxima.

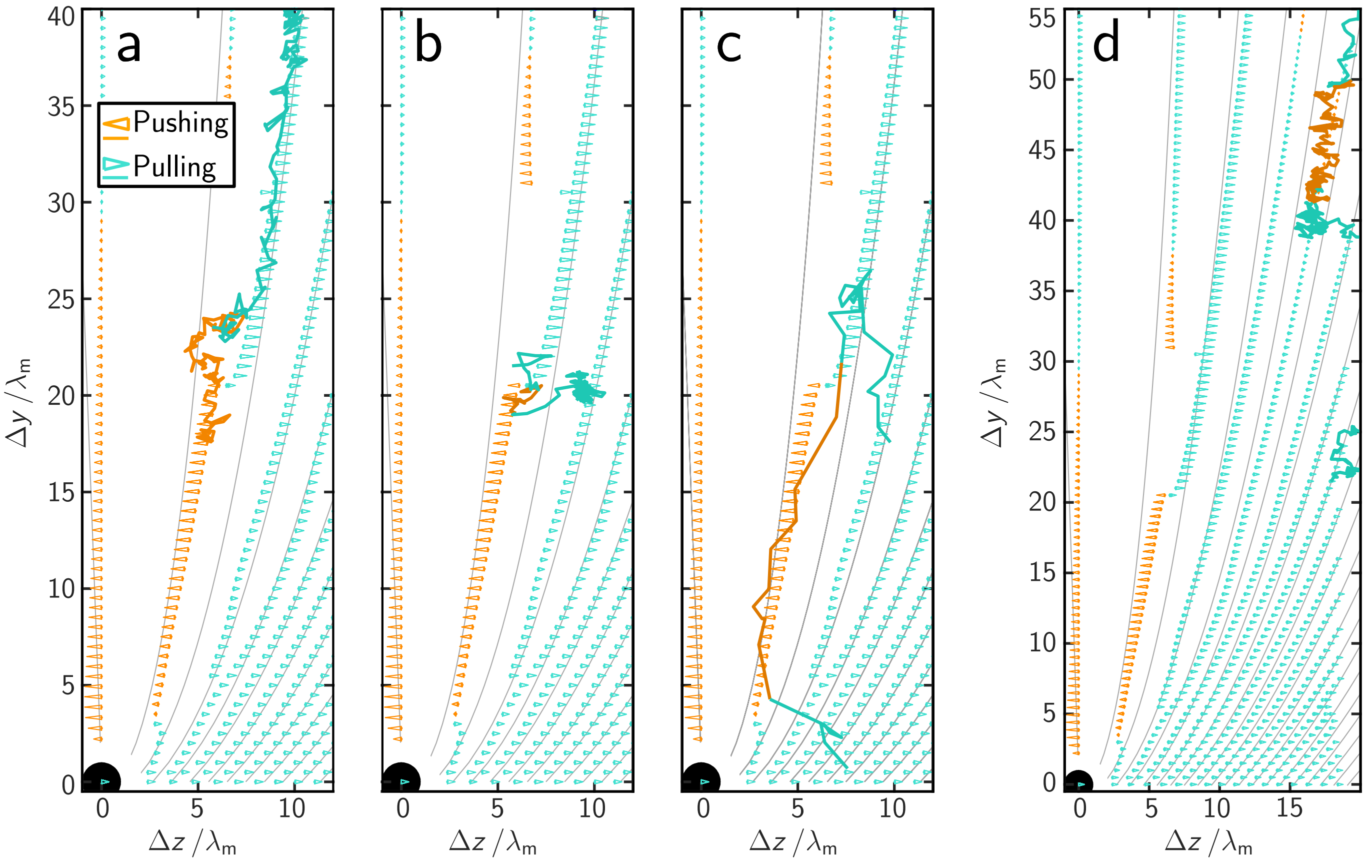

Figure S1: Comparison of the measured trajectories with the calculated particle velocities. The gray thin curves represent scattering-lobe maxima. Calculated velocities of the particle pair along the z–axis are encoded in the length of the triangles (proportional to $\sqrt{v_{\mathrm{pair}}}$). For comparison the velocity of an isolated particle v_{isol} is shown at the center of particle 1 placed at the origin of the system of coordinates. The solid zigzag curves represent experimental results and their colors encode the direction of the particle pair motion. ($\lambda_{\mathrm{m}} = 400\,\mathrm{nm}$, incident angle of the S-polarized tractor-beam $\alpha = 2.15\,^{\circ}$, polystyrene particles with 820-nm diameter).

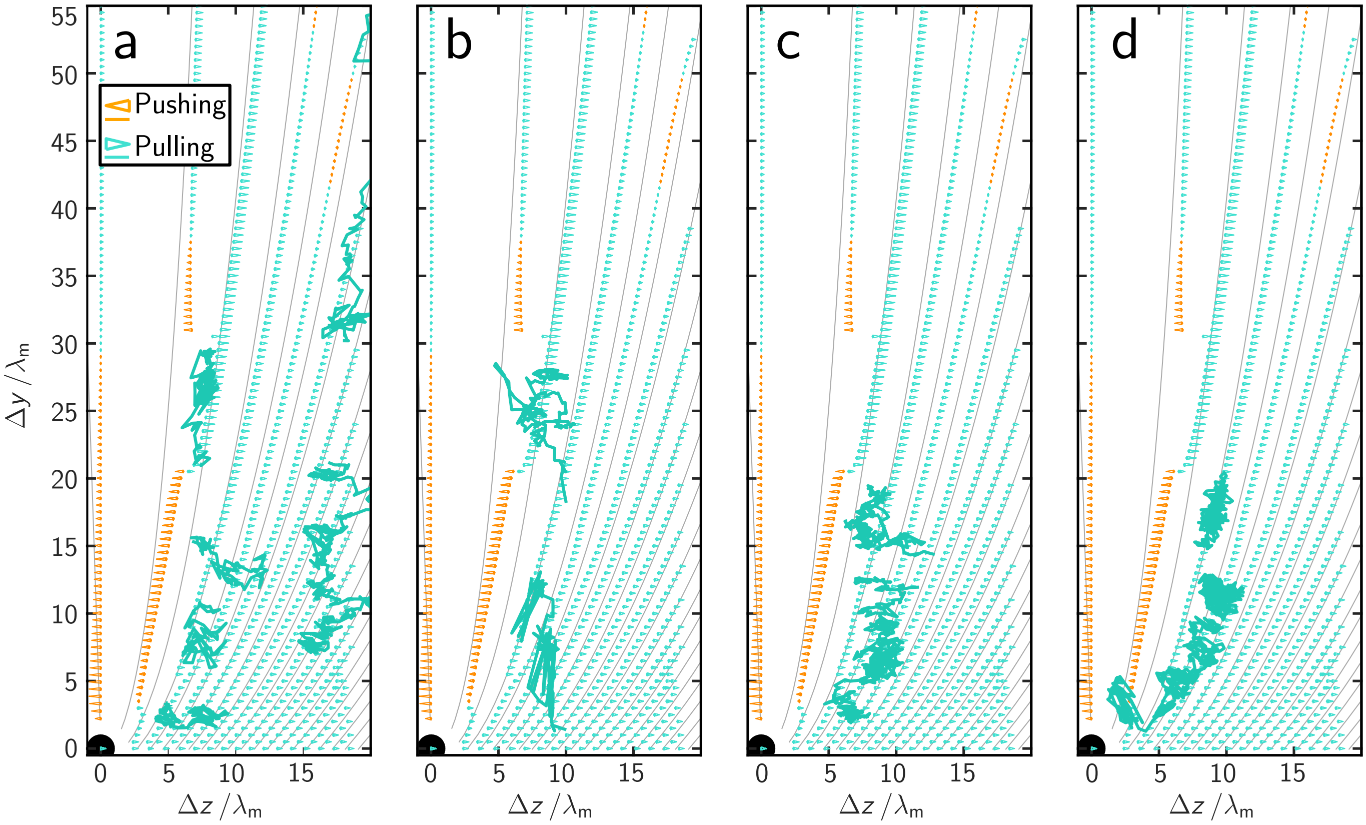

Figure S2: Comparison of the measured trajectories with the calculated particle velocities. The gray thin curves represent scattering-lobe maxima. Calculated velocities of the particle pair along the z–axis are encoded in the length of the triangles (proportional to $\sqrt{v_{\text{pair}}}$). For comparison the velocity of an isolated particle v_{isol} is shown at the center of particle 1 placed at the origin of the system of coordinates. The solid zigzag curves represent experimental results and their colors encode the direction of the particle pair motion. ($\lambda_{\text{m}} = 400\,\text{nm}$, incident angle of the S-polarized tractor-beam $\alpha = 2.15\,^{\circ}$, polystyrene particles with 820-nm diameter).

2 Theoretical description of electric-field extremes in scattering lobes

Let us assume a simplified geometry of two interfering plane waves whose wavevectors form an angle $\pi - 2\alpha$. This geometry ignores the reflection at the mirror but it is equivalent to the geometry used in the experiment for particles placed far from the mirror. Let us denote

$$k_x = 0, \tag{1}$$
$$k_y = k\cos\alpha, \tag{2}$$
$$k_z = k\sin\alpha, \tag{3}$$

where $k = 2\pi/\lambda_\mathrm{m}$, α and λ_m have the same meaning as in the main text. Assuming the wavevectors pointing *against* the z-axis (see wavevectors $\mathbf{k_1}$ and $\mathbf{k_2}$ in Figure 1a in the main text)

$$\mathbf{k}_1 = [0, -k_y, -k_z] = [0, -k\cos\alpha, -k\sin\alpha], \tag{4}$$
$$\mathbf{k}_2 = [0, +k_y, -k_z] = [0, +k\cos\alpha, -k\sin\alpha], \tag{5}$$

we can write for the interference incident field of the S-polarized tractor-beam:

$$E_{x\mathrm{i}} = E_0 \exp[i(-k_z z + k_y y)] + E_0 \exp[i(-k_z z - k_y y)] = 2E_0 \cos(k_y y)\exp(-ik_z z). \tag{6}$$

For a general direction θ in the zy-plane ($\tan\theta = z/y$) and few wavelengths away from the scattering particle, the scattered field is well described as a propagating spherical wave

$$E_{x\mathrm{s}} = E_0 \frac{F_\mathrm{S}(\theta)}{kr}\exp[i(kr + \phi_\mathrm{S}(\theta)], \tag{7}$$

where real functions F_S and ϕ_S characterize the scattering of both incident plane waves. The total electric field energy density yields

$$\begin{aligned} |E|^2 &= |E_{x\mathrm{i}} + E_{x\mathrm{s}}|^2 \\ &= 4|E_0|^2\cos^2(k_y y) + 4|E_0|^2\cos(k_y y)\cos[k_z z + kr + \phi_\mathrm{S}(\theta)]\frac{F_\mathrm{S}(\theta)}{kr} + |E_0|^2\left(\frac{F_\mathrm{S}(\theta)}{kr}\right)^2 \\ &\approx 4|E_0|^2\cos(k_y y)\left[\cos(k_y y) + \cos[kr + \phi_\mathrm{S}(\theta) + k_z z]\frac{F_\mathrm{S}(\theta)}{kr}\right]. \end{aligned} \tag{8}$$

Let us set the particle position to the maximum of the fringe along the y-axis assuming $k_y y = \pi n$ with an integer n. The scattering amplitude factor $F_\mathrm{S}(\theta)$ is positive and changes slower with a position than the cosine terms. We may therefore state the condition for the constructive interference of the two terms as

$$kr + \phi_\mathrm{S}(\theta) + kz\sin\alpha = \pi m, \tag{9}$$

where m is another integer, with even/odd values corresponding to the constructive/destructive interference. Let us further assume 'effectively isotropic' scattering represented by the parameter

$$b = \phi_{eff}/\pi. \tag{10}$$

Using $(kr)^2 = k^2(z^2+y^2) = (kz)^2 + (\pi n/\cos\alpha)^2$, we solve for the coordinate z and obtain

$$z = \frac{\lambda_{\mathrm{m}}}{2\cos^2\alpha}\left[(b-m)\sin\alpha \pm \sqrt{(m-b)^2-n^2}\right]. \tag{11}$$

Going to either side from the main forward-scattering axis, we index the consecutive lobes of decreasing intensity by integer index p. At the in-fringe maxima $p = 0, 2, 4, 6\ldots$ and at minima $p = 1, 3, 5, \ldots$

A comparison with the exactly calculated field distribution revealed that the value $\phi_{eff} = \pi/2$ gives a very good coincidence if the maxima/minima corresponding to a given lobe lie on curves defined by Eq. (11) and a constriction $m = n + p$ is valid. It gives

$$z_p = \frac{\lambda_{\mathrm{m}}}{2\cos^2\alpha}\left[(b-n-p)\sin\alpha \pm \sqrt{(2n+p-b)(p-b)}\right], \tag{12}$$

which was used to draw the electric field density maxima in Figures 2a, 2b, 3a, 4b in the main text.

The derivation of the P-polarized tractor-beam is analogical; for the external field of the tractor-beam we can write

$$\begin{aligned}\mathbf{E}^e &= E_0\begin{pmatrix}0\\ +\sin\alpha\\ \cos\alpha\end{pmatrix}\exp[i(-k_z z + k_y y)] + E_0\begin{pmatrix}0\\ -\sin\alpha\\ \cos\alpha\end{pmatrix}\exp[i(-k_z z - k_y y)]\\ &= 2E_0\exp(-iz\sin\alpha)\begin{pmatrix}0\\ i\sin(k_y y)\sin\alpha\\ \cos(k_y y)\cos\alpha\end{pmatrix}\end{aligned} \tag{13}$$

and the corresponding scattered field reads

$$\mathbf{E}^s = E_0\begin{pmatrix}0\\ -\sin\theta\\ \cos\theta\end{pmatrix}\frac{F_{\mathrm{P}}(\theta)}{kr}\exp[i(kr+\phi_{\mathrm{P}}(\theta))]. \tag{14}$$

The field energy density, after neglecting the term $\propto F_{\mathrm{P}}^2$, is

$$\begin{aligned}|\mathbf{E}|^2 \approx\ & 4E_0^2\left[\sin^2(k_y y)\sin^2\alpha + \cos^2(k_y y)\cos^2\alpha\right]\\ &+ 4E_0^2\frac{F_{\mathrm{P}}(\theta)}{kr}\left[\cos(k_y y)\cos\alpha\cos\theta\cos\Phi - \sin(k_y y)\sin\alpha\sin\theta\sin\Phi\right],\end{aligned}$$

where

$$\Phi = kr + \phi_{\mathrm{P}}(\theta,\alpha) + kz\sin\alpha. \tag{15}$$

The small value of α reduces the terms with $\sin\alpha$. Also, for θ in the narrow interval of interest, $\phi_{\mathrm{P}}(\theta) \approx \phi_{\mathrm{S}}(\theta)$ and we may well use the arguments for the case of S-polarization and employ Eq. (12).

3 Stable configurations of optically bound pairs

During calculation of optical forces, we solve the electromagnetic scattering problem in a self-consistent way, i.e., we take into account enough reflections to achieve numerical convergence. If the spheres are separated by several wavelengths, multiple scattering between spheres of a degree higher than 2 contributes negligibly to the total optical force. Thus the optical force acting on sphere 2 is determined mainly by the interaction with the field scattered only once from sphere 1 and superposed with the external incident tractor-beam field.

Let us assume that sphere 1 is held fixed in a fringe, generally with some external nonzero force. It turns out that the stable positions of sphere 2 (i.e. static equilibrium, where $F_{2,z} = 0$) emerge preferentially very close to the maxima of the scattering lobes formed by the incident field and the field scattered by sphere 1 alone, as illustrated by the green markers in Figures S3 almost overlapping with white curves denoting the intensity maxima of the lobes (following Eq. 12). Conversely, if sphere 2 is held fixed in a fringe, sphere 1 would take up some equilibrium position close to the intensity maxima arising from the light scattered by sphere 2. An intuitive picture of this behavior is illustrated in Figures 2c-h in the main text, where we show the dependence of the optical force acting on one particle on the external electric field profile and the particle position while the second particle is held fixed. However, with respect to the mirror symmetry in the xy-plane, the conditions for the static equilibrium (i.e. $F_{1,z} = F_{2,z} = 0$ giving $v_{1,z} = v_{2,z} = 0$) can be fulfilled simultaneously for both spheres only for the counter-propagating plane waves, i.e., $\alpha = 0$, $k_z = 0$.

Any nonzero $\alpha > 0$ breaks the aforementioned symmetry and stable configurations with constant inter-particle distances arise only if both spheres move uniformly with the same velocity, implying equal forces acting upon both particles $F_{2,z} = F_{1,z}$. This case is presented in Figures S3 in the form of orange (pushing) and blue (pulling) dots for the S-polarized tractor-beam. Comparing green and orange/blue dots one finds that there are much more stable moving configurations of the pair of particles. These configurations are mainly in the intensity maxima of the lobes but there are also some exceptions violating this rule. This behavior also indicates that the moving stable configurations arise as a balance between mutual demands of each particle to reach the intensity lobe maxima created by the other sphere. Figures S4 compare the pushing and pulling behavior of the moving stable configurations for both S- and P-polarizations and different incident angles α.

4 Hydrodynamical interaction between particles in a moving pair

The observed motion of a pair of particles is slow enough to give low Reynolds number $Re \approx 10^{-6} - 10^{-4}$. Thus the inertial terms can be omitted in appropriate equations of motion and the

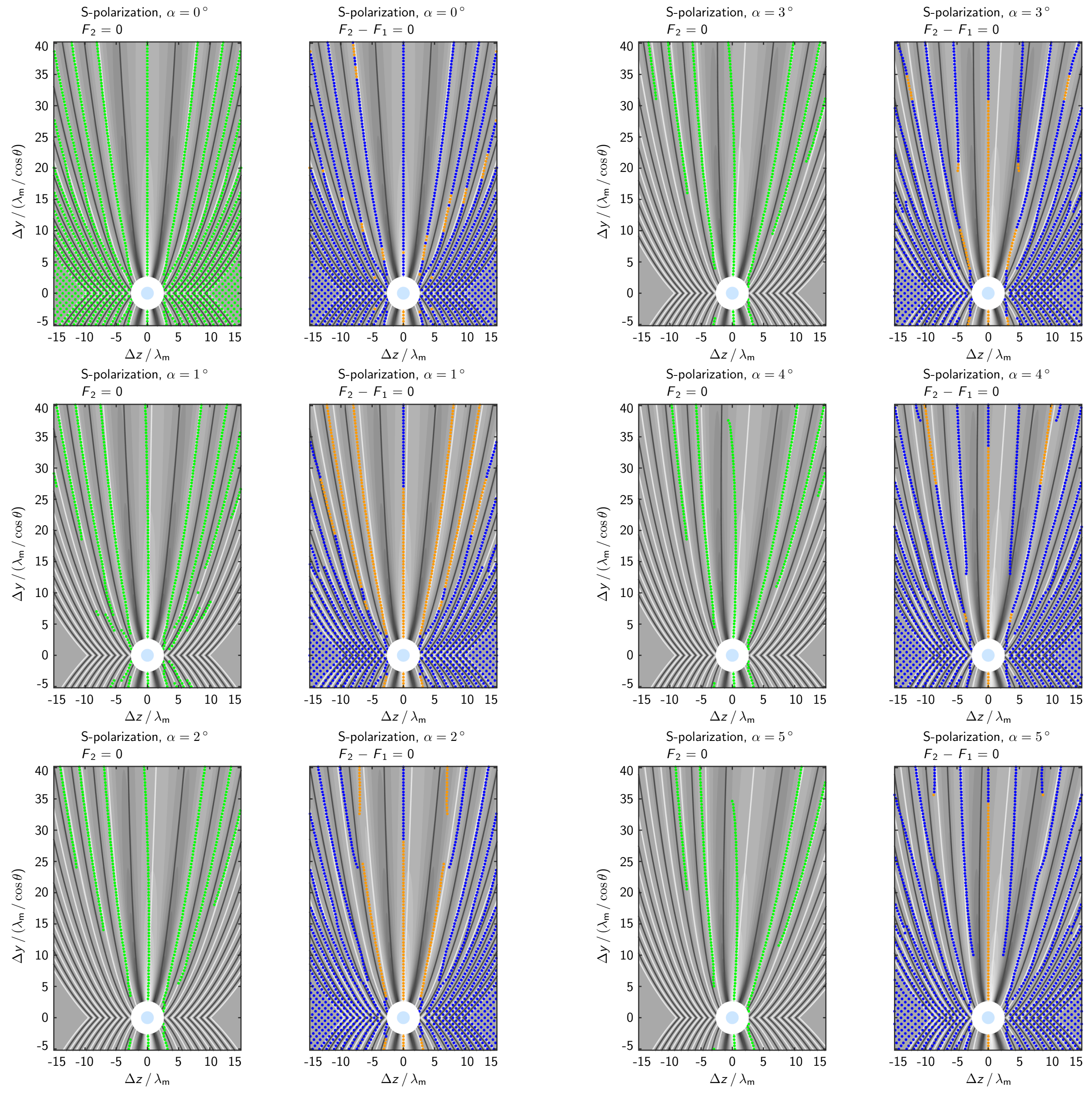

Figure S3: Comparison of static (green) and moving stable (orange and blue) configurations of a pair of particles for different incident angles α. Green dots denote the stable position of particle 2 (i.e. $F_{2,z} = 0$) if particle 1 (light larger blue dot) is kept fixed at maximum of the interference fringe along the y-axis. Orange and blue dots denote stable positions of particle 2, where both particles move with the same velocity, i.e., $F_{2,z} = F_{1,z}$ and $F_{2,y} = F_{1,y}$. The background map shows electric field energy density $|E|^2$ (in log scale) as in Figure 3a of the main text. White and gray curves mark the intensity maxima and minima of $|E|^2$ following the extrema of the lobes of the scattered field done by Eq. (12). The white annulus around particle 1 indicates the range, where the particles are too close to each other to plot the data properly.

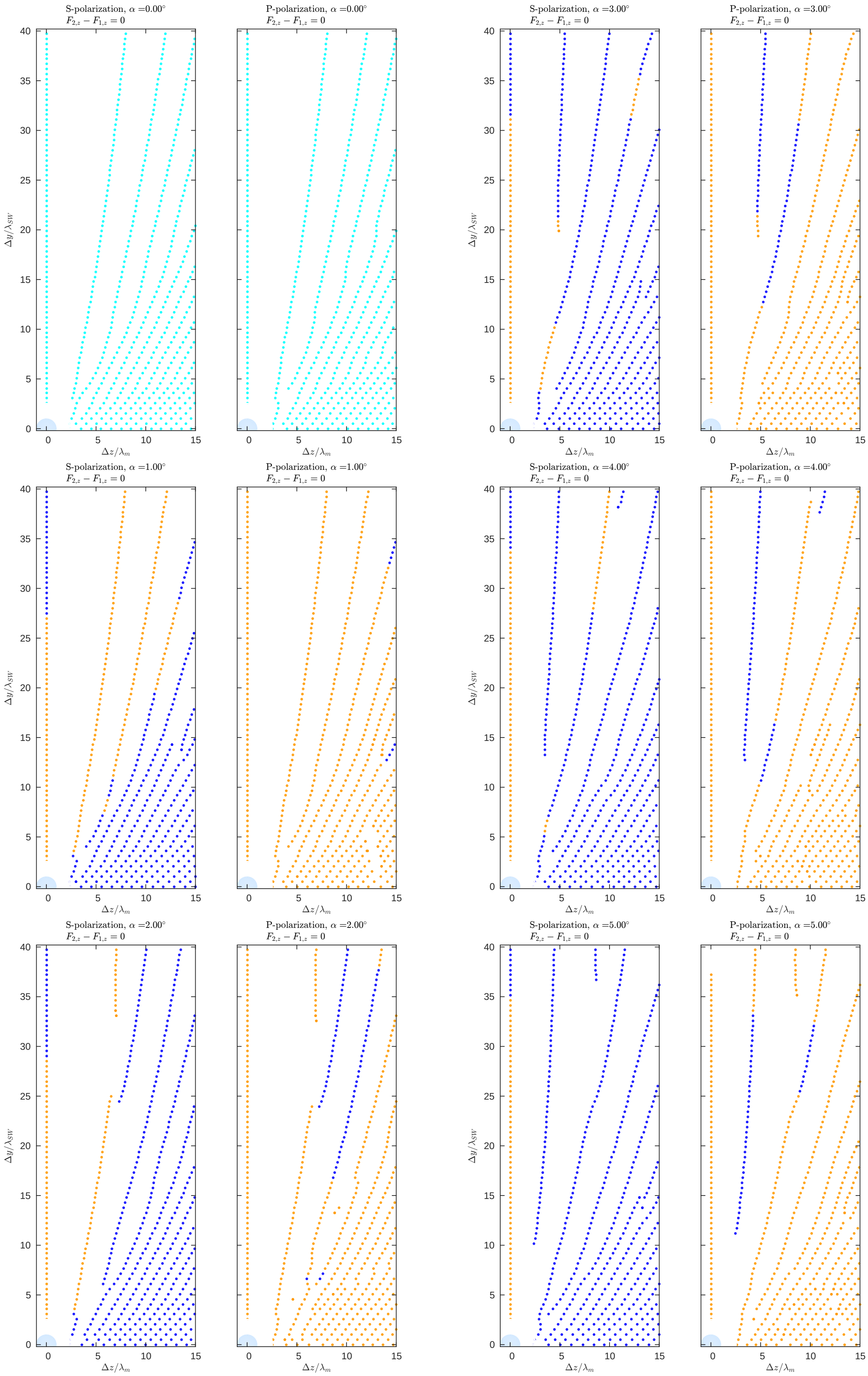

Figure S4: Theoretical results of pushing (orange) and pulling (blue) behavior of the moving stable pairs of particles for both S- and P-polarizations and different incident angles α (other parameters same as mentioned in the main text).

particle velocities are proportional to the forces as

$$\begin{pmatrix} \mathbf{v}_1 \\ \mathbf{v}_2 \end{pmatrix} = \begin{pmatrix} \boldsymbol{\mu}_{11} & \boldsymbol{\mu}_{12} \\ \boldsymbol{\mu}_{21} & \boldsymbol{\mu}_{22} \end{pmatrix} \cdot \begin{pmatrix} \mathbf{F}_1 \\ \mathbf{F}_2 \end{pmatrix}, \tag{16}$$

where $\mathbf{v}_{1,2}$ denotes the velocity of each particle, $\mathbf{F}_{1,2}$ corresponds to external forces acting on the particles (in our case exclusively given by the optical forces), and $\boldsymbol{\mu}_{ab}$ are 3×3 mobility tensors. These tensors can be split into a longitudinal and transverse component[1] as

$$\boldsymbol{\mu}_{ab,ij} = (\boldsymbol{\mu}_{\perp})_{ab,ij}(\mathbb{E} - \mathcal{P}) + (\boldsymbol{\mu}_{\|})_{ab,ij}\,\mathcal{P}, \tag{17}$$

where $\mathbb{E}$ is the identity matrix, $\mathcal{P} = \mathbf{n}^T\mathbf{n}$ is the projection matrix, $\mathbf{n}$ is the unit vector in a direction of a sphere connecting line, $i, j \in \{x, y, z\}$ denote the coordinates and $a, b \in \{1, 2\}$ index of the particle. The sphere centers are separated by a center-to-center distance $\mathbf{r} = r\mathbf{n}$.

Assuming spheres of the equal radius a, the normalized tensor components expressed up to the seventh order in $t = a/r$ [1] are the following

$$\begin{aligned}
\hat{\boldsymbol{\mu}}_{\|}{}^{11}/\mu_0 &= 1 - \frac{15}{4}t^4 + \frac{13}{4}t^6, \\
\hat{\boldsymbol{\mu}}_{\perp}{}^{11}/\mu_0 &= 1 - \frac{17}{16}t^6, \\
\hat{\boldsymbol{\mu}}_{\|}{}^{12}/\mu_0 &= \frac{3}{2}t - t^3 + \frac{75}{4}t^7, \\
\hat{\boldsymbol{\mu}}_{\perp}{}^{12}/\mu_0 &= \frac{3}{4}t + \frac{1}{2}t^3,
\end{aligned} \tag{18}$$

where $\mu_0 = 1/6\pi\eta a$ is the Stokes mobility of an isolated sphere and η the medium viscosity. The remaining components follow the relations $\boldsymbol{\mu}_{11} = \boldsymbol{\mu}_{22}$, and $\boldsymbol{\mu}_{12} = \boldsymbol{\mu}_{21}$. The condition for the sustainable uniform motion of the pair $\mathbf{v}_1 = \mathbf{v}_2 \equiv \mathbf{v}$ implies $\mathbf{F}_1 = \mathbf{F}_2 \equiv \mathbf{F}$ and

$$\mathbf{v} = (\boldsymbol{\mu}_{11} + \boldsymbol{\mu}_{12})\mathbf{F} \equiv \mathbf{M}\mathbf{F}, \tag{19}$$

where we introduced an auxiliary 'pair-mobility' tensor $\mathbf{M}$.

Let us assume the particle pairs are constrained to the yz-plane and their orientations are parametrized by an angle β, where $\mathbf{n} = (0, \cos\beta, \sin\beta)$. The velocity with a just single nonzero component along z requires nonzero F_z as well as F_y, given by equations

$$\begin{aligned}
v_z &= (M_{\|}\sin^2\beta + M_{\perp}\cos^2\beta)F_z + \sin\beta\cos\beta(M_{\|} - M_{\perp})F_y, \\
v_y \equiv 0 &= \sin\beta\cos\beta(M_{\|} - M_{\perp})F_z + (M_{\|}\cos^2\beta + M_{\perp}\sin^2\beta)F_y.
\end{aligned} \tag{20}$$

Substituting for F_y we obtain

$$v_z = \left[M_{\|}\sin^2\beta + M_{\perp}\cos^2\beta + \frac{[(M_{\|} - M_{\perp})\sin\beta\cos\beta]^2}{M_{\|}\cos^2\beta + M_{\perp}\sin^2\beta}\right]F_z. \tag{21}$$

Figure S5 compares velocities of pairs with different inter-particle distances r and orientations β with respect to the y-axis. Keeping the mobility components only up to the first degree in t (using Oseen approximation) yields

$$v_z \approx \left[1 + \frac{3}{4}(1 + \cos^2\beta)t + \frac{9}{16}\frac{[\sin\beta\cos\beta]^2}{1 + \frac{3}{4}(1 + \cos^2\beta)}t^2\right]v_{z0}, \tag{22}$$

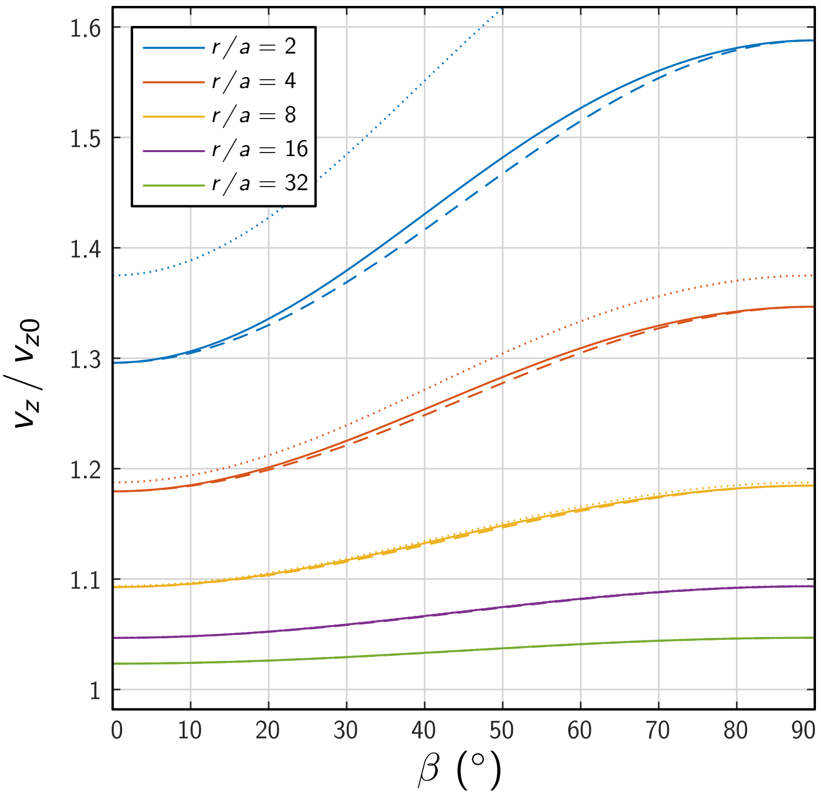

Figure S5: Speed v_z of a pair of particles of the radius a related to the speed v_{z0} of a pair with ignored hydrodynamic biding as a function of the inter-particle distance r and pair orientation β with respect to the y-axis. The dashed curves show the effect of disregarding the 'constraint' term in Eq. (21). The Ossen approximation in Eq. (22) is shown by dotted curves and it overestimates mobility for the particle in contact, but for $r/a > 8$ approximately overlaps with the higher-order result. The value $v_z/v_{z0} = 1$ corresponds to the case, where the hydrodynamic binding is ignored.

where we denoted $v_{z0} = \mu_0 F_z$ as the velocity of the pair omitting the hydrodynamic interaction. Note that the second order term t^2 is a consequence of the velocity constraint ($F_y \neq 0$). Even though this term vanishes for orientations $\beta = 0, \beta = \pi/2$, it contributes by a small amount for any other inclination. It implies that in the moving stable state, both spheres must be slightly deflected out of the fringe centers in the same direction of the y-axis.

For the particle size used in the presented experiments, the fringe stiffness would allow only a tiny fringe-transverse displacement so that the fringe-parallel forces remain almost unaffected. However, for spheres positioned close to each other and sphere sizes weakly sensitive to the standing wave fringes along the y-axis, the hydrodynamic interaction together with the binding forces may cause significant shift of the spheres out of the equilibrium position along the y-axis.

References

[1] Reuland P, Felderhof BU, and Jones RB. Hydrodynamic interaction of two spherically symmetric polymers. *Physica A* 1978; **93**: 465–475.
