# Supplementary material for: Enhancement of the ‘tractor-beam’ pulling force on an optically bound structure
Source: Light Sci Appl. 2018 Jan 12;7:17135–. doi: 10.1038/lsa.2017.135 (PMC6107043; doi:10.1038/lsa.2017.135)
Supplement: Supplementary Movies [file lsa2017135x2.pdf]

Movie 1

Recorded video of two optically bound particles (polystyrene spheres with 820-nm diameter) with background subtracted. Parameters used during the experiment: incident angle of the S-polarized tractor-beam $\alpha = 2.15\,^{\circ}$, power in the sample plane $P = 3.5\,\mathrm{W}$, beam waist $w_0 = 35\,\mu\mathrm{m}$, frame rate 100 frames/s.

Movie 2 and 3

An intuitive picture of the optical interaction between two particles via scattered light interfering with the incident tractor-beam in the form of lobes if the particle pair is pulled or pushed. The left plots show the electric field energy density in y sections of two particles along the z–axis (filled green/red), electric field gradient (green/red line) and the optical forces (black line) acting on the particular particle, which is drawn at the position corresponding to the stable equilibrium configuration of the particle pair (i.e., the binding force between particles equals zero). The other plots show stable positions of optically bound pair in the tractor-beam, localized very close to interfering lobe maxima (marked with solid curves). The right plot additionally show optical forces acting on the second particle. The unfilled black arrows represent the tractor-beam force acting on an isolated particle, the filled arrows indicate the interaction gradient force caused by the field modification by the first particle. Parameters used in numerical calculations: $\lambda_{\mathrm{m}} = 400\,\mathrm{nm}$, incident angle of the tractor-beam $\alpha = 2.15\,^{\circ}$, polystyrene particles with 820-nm diameter. Movie 2 is valid for S-polarized tractor-beam, Movie 3 for P-polarized tractor-beam.
